# In Reply

**DOI:** 10.1093/oncolo/oyac171

**Published:** 2022-08-29

**Authors:** Mario E Lacouture, Anisha B Patel, Jonathan E Rosenberg, Peter H O’Donnell

**Affiliations:** Department of Medicine, Memorial Sloan Kettering Cancer Center, New York, NY, USA; Weill Cornell Medical Center, New York, NY, USA; Department of Dermatology, Internal Medicine, The University of Texas MD Anderson Cancer Center, Houston, TX, USA; Department of Medicine, Memorial Sloan Kettering Cancer Center, New York, NY, USA; Weill Cornell Medical Center, New York, NY, USA; Department of Medicine, Section of Hematology/Oncology, University of Chicago, Chicago, IL, USA

## Abstract

This letter to the editor responds to remarks on a recently published article about dermatologic events associated with enfortumab vedotin and agrees that education on the prevention and management of such events should be a priority.

We appreciate the letter from Dr. Ingen-Housz-Oro et al^1^ about our recently published article^2^ and agree education on the prevention and management of skin reactions that can occur with enfortumab vedotin should be a priority. Early recognition, accurate diagnosis, appropriate dose modifications, and when appropriate, consultation with specialists are key.^3-5^ The impetus for our manuscript was to further educate health care providers on knowledge and best practice recommendations to anticipate and manage these potential adverse events.

Enfortumab vedotin is the first and only therapy known to improve overall survival, compared with chemotherapy, in locally advanced/metastatic urothelial cancer (la/mUC) in the post platinum-based chemotherapy and post-PD-1/PD-L1 inhibitor setting. As such, the role of appropriate adverse event management to allow patients with limited treatment options to receive enfortumab vedotin is critical to the safe use of this drug. These strategies have also been further contextualized by dermatologists and oncologists with relevant experience managing dermatologic events. We believe these practice recommendations have the potential to improve quality of life and patient outcomes.

Indeed, there is a spectrum of dermatologic manifestations observed with enfortumab vedotin, given that its target, Nectin-4, is normally expressed at the surface of keratinocytes and epithelium of sweat glands and hair follicles.^2^ The referenced cases from Dr. Ingen-Housz-Oro and colleagues^1^ further highlight the importance of close monitoring for signs and symptoms as early as the first cycle (when skin reactions often present), the use of recommended dose modifications, including dose holds, and intervention for patients with these reactions receiving enfortumab vedotin. Close collaboration with a dermatologist is also recommended. All of this guidance is detailed further in the manuscript.^2^

We agree that further research is important. Several clinical trials to investigate enfortumab vedotin in first-line treatment of la/mUC, in muscle-invasive bladder cancer, and in other solid tumors are ongoing. Data from clinical trials, combined with observations from case reports and clinical management manuscripts, will allow refinement of best practice recommendations for optimizing the mitigation and management of skin reactions with enfortumab vedotin, both alone and in combination with other agents.
